# Effect of periodontal therapy on endothelial function and serum biomarkers in patients with periodontitis and established cardiovascular disease: a pilot study

**DOI:** 10.3389/froh.2025.1488941

**Published:** 2025-02-10

**Authors:** Ana Molina, Nagore Ambrosio, María Molina, Eduardo Montero, Leire Virto, David Herrera, Elena Figuero, Mariano Sanz

**Affiliations:** ^1^ ETEP (Etiology and Therapy of Periodontal and Peri-implant Diseases) Research Group, University Complutense, Madrid, Spain; ^2^ Unit of Cardiac Rehabilitation, Department of Cardiology, Hospital Universitario Severo Ochoa, Leganés, Spain

**Keywords:** periodontitis, periodontal therapy, cardiovascular diseases, flow-mediated dilation, carotid intima-media thickness

## Abstract

**Aim:**

To investigate the effect of periodontal therapy on endothelial function of subjects with periodontitis in stages III or IV and established cardiovascular disease.

**Materials and methods:**

A triple-blinded, parallel groups, randomized clinical trial of 6 months duration, on patients with history of coronary heart disease and periodontitis in stages III or IV was performed. Intervention consisted of steps 1 (oral hygiene instructions and professional mechanical plaque removal) and 2 (subgingival instrumentation) of periodontal therapy, including an antiseptic mouth rinse for 7 days. Patients in the control group received only step 1, with the adjunctive use of a fluoride-containing mouth rinse. Endothelial function (flow-mediated dilation [FMD]) and carotid intima-media thickness (cIMT) at baseline, 3 and 6 months, and serum markers of inflammation and cell adhesion at 3 days, 10 days, 3 and 6 months after therapy, were evaluated. Demographic characteristics, cardiovascular risk factors, history of cardiovascular diseases, medication intake, lipids profile, blood pressure, and periodontal outcomes were also evaluated. Student T, Mann–Whitney U, Chi-square and Fisher-exact tests were performed along with repeated measures ANOVA with *post hoc* Bonferroni's corrections.

**Results:**

Thirty-five patients were included. In the test group, improvements in pocket depth, bleeding on probing and suppuration at 6 months were significantly better than in control patients. Reductions in mean FMD [*test group* −3.43%; 95% confidence interval—CI [−2.68; 9.54], *p* = 0.487; *control group* −6.75%; 95% CI [1.29; 12.22], *p* = 0.012] and cIMT (*test group* −0.05 mm; 95% CI [0.01; 0.10], *p* = 0.014; *control group* −0.01 mm; 95% CI [−0.03; 0.05], *p* = 1.000) were observed in both groups from baseline to 6 months, without significant intergroup differences at any time-point. Differences between groups in serum inflammatory markers were detected at baseline and 3 days for interleukin (IL)-18, and at 10 days for IL-8.

**Conclusion:**

Preliminary results from the present pilot study showed that steps 1 and 2 of periodontal treatment in subjects with periodontitis in stages III–IV and established cardiovascular disease induced improvements in cIMT and periodontal outcomes, although changes in FMD were not observed.

**Clinical Trial Registration:**

clinicaltrials.gov, Identifier, database (NCT02716259).

## 1 Introduction

Cardiovascular diseases (CVD) are non-communicable conditions affecting the heart and vessels, that include ischemic heart disease, cerebrovascular disease, peripheral arterial disease, and rheumatic heart disease, among others. CVD constitute the leading cause of death worldwide (1). The majority of these diseases have in common the pathobiological process of atherosclerosis, resulting in the formation of extracellular lipid deposits in the arterial walls. Although its aetiology is not fully elucidated, atherosclerosis onset and progression is influenced by the effect of various factors, including a predisposing genetic background, hypercholesterolemia, modified lipoproteins, hypertension, diabetes mellitus, smoking and certain infections (2, 3). Furthermore, since atheroma formation is a chronic inflammatory process within the vessel wall, it is influenced by systemic chronic inflammation (2, 4).

Periodontitis is a multifactorial chronic inflammatory disease, triggered by dysbiotic changes in the subgingival biofilm, and characterized by destruction of the tooth attachment apparatus (5), coupled with a dysregulated and non-resolving inflammatory response (6). Furthermore, this localized chronic inflammation results in a low-grade systemic inflammatory status, mainly in the cases of severe periodontitis (stages III–IV) (7). The impact of severe periodontitis on systemic inflammation, resulting in increased levels of inflammatory and pro-coagulation markers in serum, has been hypothesized as a plausible mechanism to explain the association of periodontitis with the pathobiology of atherosclerosis (7–10). Furthermore, certain oral bacteria, including some well-known periodontal pathogens, have shown capability to access the bloodstream and invade the inner walls of the vessels, thus hypothetically inducing and promoting the atherosclerotic lesion, as it has been demonstrated in mechanistic investigations in animal models (9, 11). Evidence from epidemiological studies have linked periodontitis with a higher risk of future CVD (12), subclinical atherosclerosis (13–15), established CVD (including coronary heart disease, stroke, and peripheral arterial disease) (16) and premature death from all causes and from CVD (17).

Current treatment of periodontitis is based, not only on removal of the subgingival biofilm deposits and associated bacteria, but also on risk factor control mainly through smoking cessation interventions (18). Beyond its effects in arresting periodontal inflammation, periodontal therapy has proven to be efficient in reducing systemic inflammation (7) and in improving certain surrogate measures of CVD on healthy subjects and in patients with high risk of CVD (9, 19). However, similar evidence on subjects with established CVD is limited and inconclusive (20–22).

It was, therefore, the objective of this pilot clinical trial to investigate the effect of steps 1 and 2 of periodontal therapy on the endothelial function (measured by flow-mediated dilation) in patients with periodontitis in stages III or IV and established CVD. As secondary objectives, the effect of periodontal therapy on periodontal parameters, carotid intima-media thickness, and serum levels of coagulation and systemic inflammation markers were evaluated.

## 2 Materials and methods

### 2.1 Study design

This study was designed as a parallel-group, triple-blinded (patient, examiner and statistician) randomized clinical trial (RCT), with a 6-month follow-up, in patients with periodontitis in stages III or IV, and established CVD. The study protocol was designed in accordance with the Helsinki Declaration (2008) and it was approved by the Ethics Committee of Complutense University (Madrid, Spain) and Severo Ochoa University Hospital (Leganés, Madrid, Spain). The study protocol was registered at *clinicaltrials.gov* database (NCT02716259).

### 2.2 Sampling

Participants were screened among patients attending the Unit of Cardiac Rehabilitation, at the Department of Cardiology of Severo Ochoa University Hospital in Leganés, Spain. All patients gave written informed consent prior to inclusion in the trial.

### 2.3 Inclusion criteria

Patients had to be ≥18 years old and to present established CVD, i.e., history of a coronary acute syndrome (myocardial infarction or angina) in the past 3–12 months and a left ventricular ejection fraction ≥50%. Patients included had a diagnosis of periodontitis in stages III or IV according to the 2017 World Workshop on the Classification for Periodontal and Peri-implant Diseases and Conditions (5), with probing depths (PD) >5 mm and marginal bone loss >30% in ≥50% of their teeth, and a minimum of three teeth per quadrant.

Exclusion criteria were:

• Periodontal treatment in the previous 12 months.
• Antibiotic intake in the previous 3 months.
• Smokers of ≥10 cigarettes per day.
• Pregnant or nursing women.
• Diabetes mellitus type 1, or type 2 with HbA1c >7.
• HIV infection.
• Chronic use of non-steroid anti-inflammatory drugs.
• Necrotizing periodontal diseases.

### 2.4 Intervention

**Figure 1:**
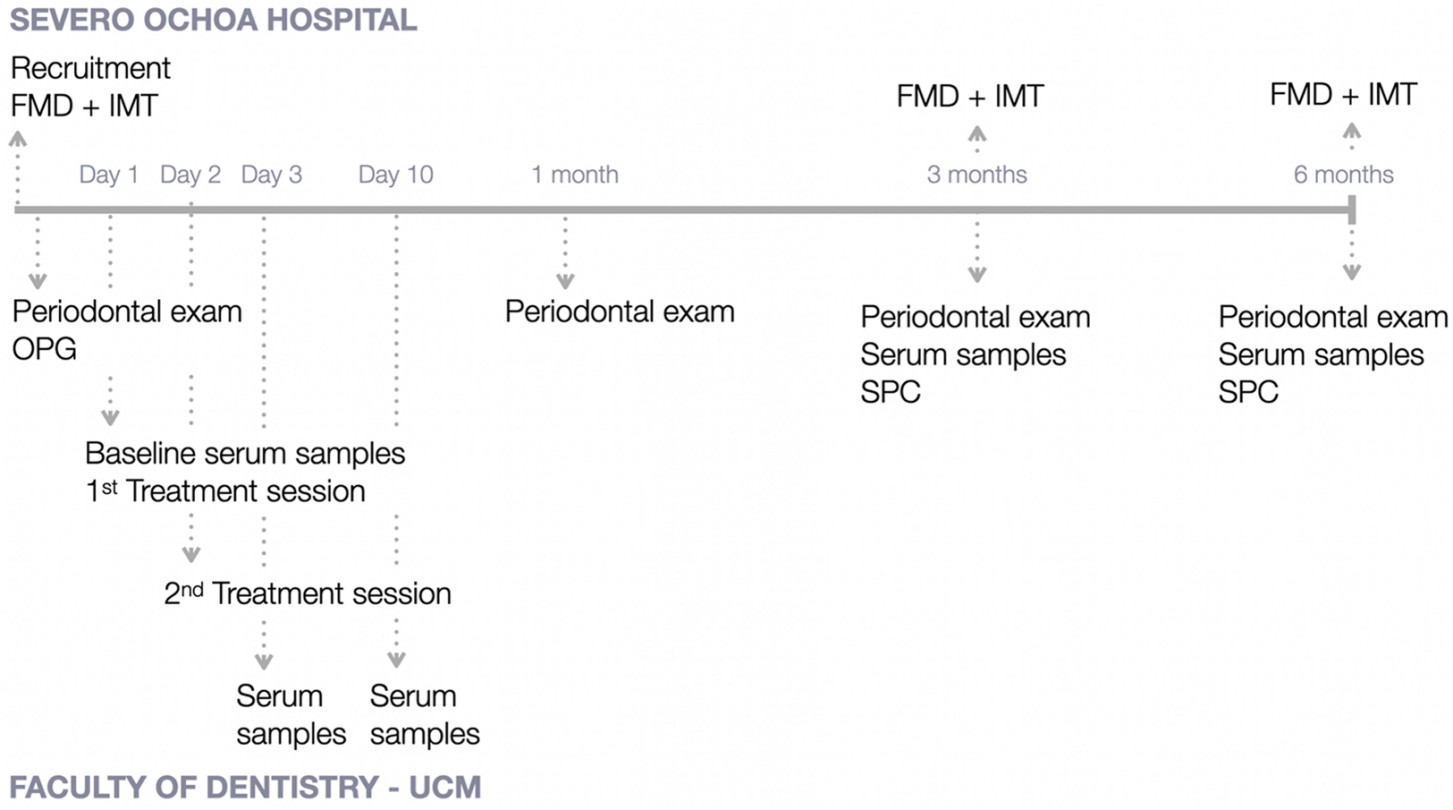
Study outline. FMD, flow-mediated dilation; IMT, intima media thickness; OPG, orthopantomography; SPC, supportive periodontal care; UCM, University Complutense of Madrid.

Figure 1 depicts the study outline, with the intervention and measures recorded at every time point at Severo Ochoa University Hospital and at the Postgraduate Clinic in Periodontology at Complutense University.

After being included in the trial and once the baseline examinations were performed, patients were randomized to receive either subgingival instrumentation with ultrasonic devices, curettes and prophylaxis paste (within step 2 of periodontal therapy) with adjunctive use of a 0.12% chlorhexidine (CHX) and 0.05% cetylpyridinium chloride (CPC) mouth rinse (PerioAid® Tratamiento, Dentaid, Barcelona, Spain) for 7 days (test group), or professional mechanical plaque removal (PMPR) with ultrasonic devices, curettes and prophylaxis paste (within step 1 of periodontal therapy) with adjunctive use of a fluoride mouth rinse (FluorAid®, Dentaid, Barcelona, Spain) for 7 days (control group), in two consecutive sessions under local anaesthesia.

Randomization was prepared by an independent researcher using block randomization (size of the block = 6). Allocation concealment was kept by means of opaque sealed envelopes, and randomization was revealed to the operator immediately before starting the intervention.

All procedures were performed by a single trained periodontist (AM). Subjects in both groups received step 1 of periodontal therapy, including oral hygiene instructions (modified Bass brushing technique and the use of interdental brushes). In follow-up visits (3- and 6-month), supportive periodontal care (SPC) was provided by the same operator, consisting of oral hygiene reinforcement and PMPR with ultrasonic devices, curettes and prophylaxis paste and subgingival instrumentation, if appropriate, in the test group, and oral hygiene reinforcement and PMPR alone in the control group.

### 2.5 Measures

#### 2.5.1 Demographic and medical parameters

Sociodemographic characteristics were collected at baseline by patient interview and included age, gender, smoking habit (never smoker, ex-smoker, current smoker), high level of stress (yes/no), sedentarism (yes/no), postmenopausal (yes/no), and family history of premature CVD (yes/no). Medical parameters were obtained from the hospital records of the participants, at baseline and at 6 months follow-up, including: allergies, history of other systemic diseases (besides CVD), medication intake, total cholesterol, high-density lipoprotein cholesterol (HDL-C), low-density lipoprotein cholesterol (LDL-C) and triglycerides (TG). Blood pressure (BP) measurements with a digital BP machine (Omron X2 Basic, Omron Healthcare Co. Ltd., Kyoto, Japan) and anthropometric measurements [height, weight, abdominal circumference, and body mass index (BMI)] were recorded by the researchers at baseline and 6 months.

#### 2.5.2 Vascular parameters

An experienced cardiologist (MM) performed the recording of vascular parameters at baseline, 3- and 6-month of follow-up, at the Severo Ochoa University Hospital, with an ultrasound (Vivid E9, General Electric HealthCare, Horton, Norway) and a linear array transducer of 7 MHz (9l Ultrasound probe, General Electric HealthCare, Horton, Norway). Flow mediated dilation (FMD) was automatically measured (Brachial Tools, version 3.2.6, Medical Imaging Applications, Coralville, IA, USA) at the brachial artery in response to 250 mmHg arterial pressure exerted with a manual sphygmomanometer for 5 min. Dilation was calculated as the change, expressed in percentage, between baseline to the maximum dilation peak measured between 45 and 75 s after releasing the cuff pressure. Carotid intima-media thickness (cIMT) was evaluated by ultrasounds. With the patient in dorsal decubitus, images were taken from the dorsal walls of the common carotid at 1 cm from the carotid bifurcation. Maximal values of cIMT were calculated with an automatic border detection software for cIMT measurement (EchoPAC®, General Electric HealthCare, Horton, Norway).

#### 2.5.3 Periodontal parameters

Periodontal examinations were performed by a single trained periodontist (EM), blinded to the allocated treatment, at baseline, one month, three months and six months after therapy. PD, gingival recession (Rec), clinical attachment level (CAL), plaque index (PlI), bleeding on probing (BoP), and suppuration (Sup) were recorded at 6 sites in all teeth except third molars with a UNC-15 manual periodontal probe. Tooth mobility and furcation involvement were also assessed. Mean values of each periodontal variable were calculated per patient.

#### 2.5.4 Serum samples

Serum samples were taken at baseline, 3-days, 10-days, 3-months and 6-months for the quantification of serum levels of inflammatory mediators (interleukin [IL]-1β, IL-6, IL-8, IL-10, IL-18 and tumour necrosis factor [TNF]-α) and endothelium activation markers (soluble intercellular adhesion molecule-1 [sICAM-1] and soluble vascular adhesion molecule 1 [sVCAM-1]). Peripheral venous blood samples were taken from the antecubital fossa or the dorsum of the hand using an intravenous catheter (VacutainerTM, Becton, Dickinson and Company, Franklin Lakes, NJ, USA) with a conventional technique, according to the recommendations from the Spanish Society of Infectious Diseases and Clinical Microbiology (23). Samples were processed at the Microbiology Laboratory of the Faculty of Odontology at Complutense University of Madrid using high-sensitivity multiplex map human immunoassays. Laboratory tests are further explained in the Supplementary (Section 1.1).

#### 2.5.5 Adverse events

The occurrence of adverse events, either cardiovascular or treatment-related, were recorded at every study visit.

### 2.6 Data analysis

Sample size was calculated to detect a 1% difference in FMD between groups, with a standard deviation (SD) of 1.67% (19) at a two-sided alpha level of 5% and 90% power. A total of 96 patients, 48 per group were required. To compensate for possible dropouts and adjustment for confounders, a final sample of 120 was established (20% more). Since the intended sample was not achieved, a post-hoc power and sample size calculation was performed.

The primary outcome variable was the change in FMD (baseline-6 months). Secondary outcomes included all other previously described. Changes between baseline and 6 months, baseline to 3 months and 3- to 6-month visits were calculated. A subject-level analysis was performed for each study parameter. Data were expressed by means and SD (for quantitative variables), prevalence and proportions (%) (for qualitative variables). Normality of the distribution of quantitative variables were assessed by means of Shapiro-Wilk normality test and box plots. Differences between groups in quantitative variables at baseline, 3- and 6-month visits and their changes were determined by the Student *t* test or Mann–Whitney *U* test for quantitative outcomes. Additionally, repeated measures ANOVA with *post hoc* Bonferroni's correction considering the visit for the intra-group comparisons, the group (intervention or control) for the inter-group comparisons, and the interaction between time and group, was performed. Categorical data were compared by means of the Chi-square test or Fisher-exact test. The level of statistical significance was set at *p* < 0.05. A statistical software package IBM®SPSS Statistics 29.0 (IBM Corporation, Armonk, NY, USA) was used for all data analysis.

## 3 Results

### 3.1 Sample description

**Figure 2:**
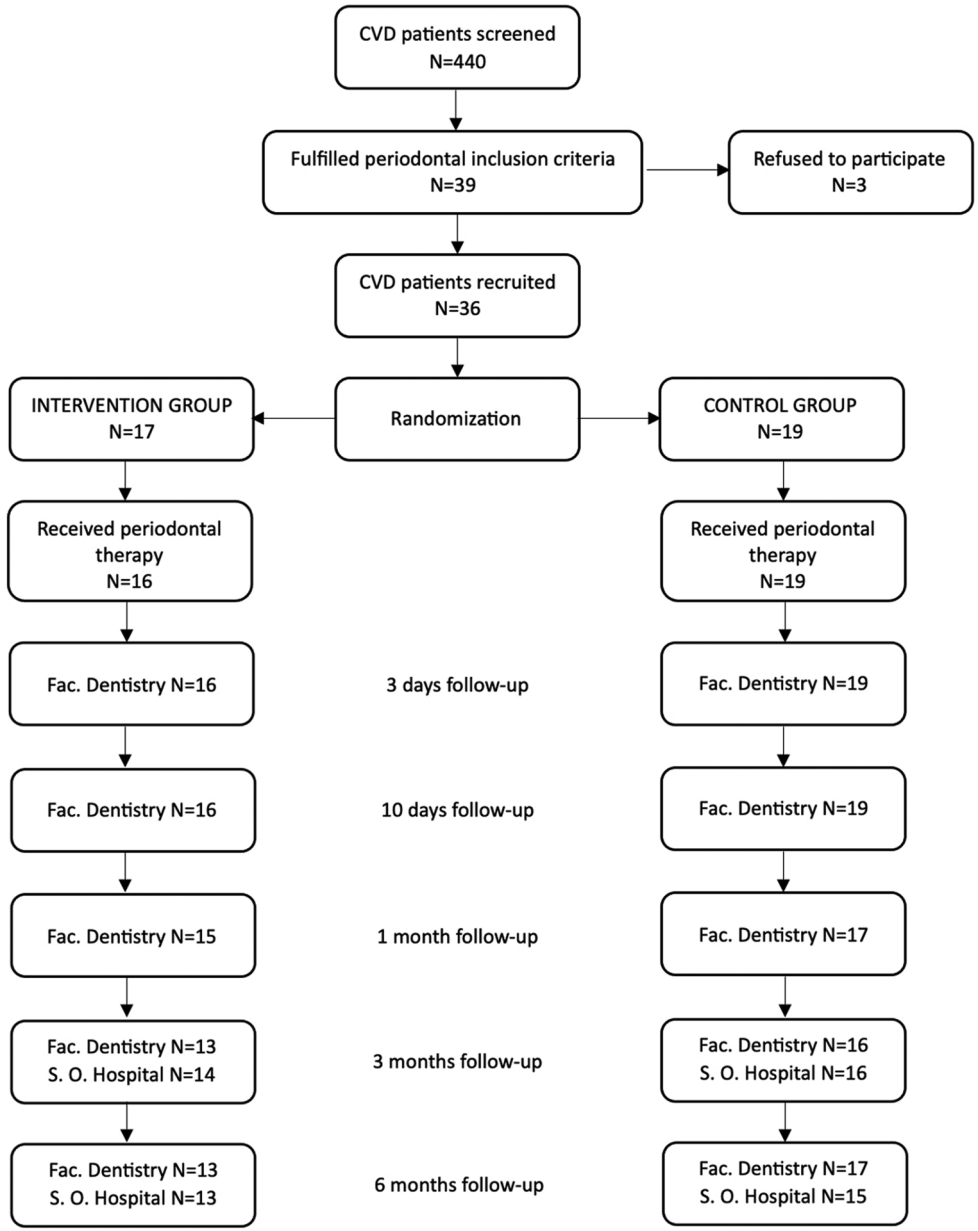
Study flow-chart. CVD, cardiovascular diseases; Fac., Faculty; S.O., Severo Ochoa.

Figure 2 depicts the flow chart of the study. A total of 440 subjects with history of coronary acute syndrome in the past 3–12 months, attending the Unit of Cardiac Rehabilitation of Severo Ochoa Hospital, were considered for inclusion. The screening period lasted from February 2016 to February 2020. Thirty-nine patients fulfilled the periodontal inclusion criteria and were invited to enter the trial; three of them refused to participate due to inability to travel to Madrid and attend the visits at the Faculty of Odontology. Finally, 36 patients were recruited and randomized; one of them could not receive the intervention assigned due to the onset of the Covid-19 pandemic, so data from 35 patients were included in the present study. Sixteen patients were randomized to test group and 19 to control group. One subject from control group was excluded at 3 months of follow-up due to having receive further periodontal treatment in a private clinic (Figure 2).

### 3.2 Demographic characteristics and cardiovascular risk factors

Baseline characteristics of the study sample are shown in Table 1. The mean age was 61.05 years in test group and 55.55 years in control group, and three participants were women (one test, two controls). Most of the participants were former smokers (75% test group, 73.7% control group), and three subjects (one test, two controls) were still smokers of <10 cigarettes per day. High stress levels were reported by 19 subjects (62.5% test group, 47.4% control group) and five subjects in each group referred to have family history of CVD (31.3% test group, 26.3% control group). Mean BMI in both treatment groups was in the over-weight category (>25).

**Table 1:** Baseline characteristics of the study sample.

	Test group	Control group	*p* value
	*n* = 16	*n* = 19	
Demographic Characteristics			
Age mean (SD)	61.05 (17.06)	55.55 (10.23)	0.247
Gender *n* (%)			
Female	1 (6.3)	2 (10.5)	1.000
Male	15 (93.8)	17 (89.5)	
Smoking status			
Never smoker *n* (%)	3 (18.8)	3 (15.8)	0.889
Former smoker *n* (%)	12 (75)	14 (73.7)	
Current smoker *n* (%)	1 (6.2)	2 (10.5)	
High stress levels *n* (%)	10 (62.5)	9 (47.4)	0.371
Postmenopause *n* (%)	0 (0)	2 (10.5)	0.489
Family history CVD *n* (%)	5 (31.3)	5 (26.3)	1.000
Sedentarism *n* (%)	7 (43.8)	7 (36.8)	0.678
BMI mean (SD)	27.19 (3.26)	27.37 (4.07)	0.833
Cardiovascular Diseases			
Peripheral arterial disease *n* (%)	1 (6.3)	0 (0)	0.206
Cerebrovascular accident *n* (%)	0 (0)	0 (0)	–
Coronary acute syndrome			
STEACS *n* (%)	7 (43.8)	6 (31.6)	0.458
Bypass treatment *n* (%)	2 (12.5)	1 (5.3)	0.582
Stent treatment *n* (%)	14 (87.5)	16 (84.2)	0.781
No vessels with 50% blockage mean (SD)	1.50 (0.63)	1.74 (0.73)	0.319
Time (months) from CVD event mean (SD)	9.93 (4.10)	8.78 (3.80)	0.398
Comorbidities			
Dyslipidaemia *n* (%)	6 (37.5)	8 (42.1)	0.782
Hypertension *n* (%)	6 (37.5)	2 (10.5)	0.105
Diabetes Mellitus *n* (%)	2 (12.5)	3 (15.8)	1.000
DM type I *n* (%)	0 (0)	0 (0)	–
DM type II *n* (%)	2 (100)	3 (100)	1.000
Depression *n* (%)	1 (6.3)	1 (5.3)	0.900
Other conditions *n* (%)	7 (43.8)	5 (26.3)	0.279
Periodontal Diagnosis			
Stage III grade A *n* (%)	0 (0)	0 (0)	0.378
Stage III grade B *n* (%)	8 (50)	7 (36.8)	
Stage III grade C *n* (%)	4 (25)	4 (21.1)	
Stage IV grade A *n* (%)	0 (0)	0 (0)	
Stage IV grade B *n* (%)	2 (12.5)	1 (5.3)	
Stage IV grade C *n* (%)	2 (12.5)	7 (36.8)	

SD, standard deviation; CVD, cardiovascular diseases; BMI, body mass index; STEACS, ST-segment elevation acute coronary syndrome; *n*, number; DM, diabetes mellitus.

Regarding cardiovascular disease diagnosis, all subjects had suffered a coronary acute syndrome 3–12 months prior to study inclusion and presented with left ventricular ejection fraction ≥50%. Most of the participants (87.5% test group, 84.2% control group) had received reperfusion therapy (stent placement) in one or more coronary arteries, and three participants had undergone bypass surgery (12.5% test group, 5.3% control group). ST-segment elevation acute coronary syndrome (STEACS) had occurred in seven (43.8%) subjects in the test and six (31.6%) subjects in the control group; one subject in the test group had also been diagnosed with peripheral arterial disease, and none had suffered a cerebrovascular accident. Other comorbidities, diagnosed prior to the onset of the CVD event, such as dyslipidaemia and hypertension, were frequent.

Medication intake at baseline and 6 months is summarised in Supplementary Table S1. In the selected sample population, all participants were under treatment with a minimum of three drugs: one antithrombotic, one hypolipidemic and one antihypertensive drug. At baseline, most subjects were under the combination of two antiplatelet drugs (81.3% test group, 89.5% control group), and four of them were also prescribed anticoagulant medication (acenocumarol) (18.8% test group, 5.3% control group). Patients were prescribed one to four antihypertensive medications, and eight subjects used vasodilators (25% test group, 21.1% control group). Significant differences between groups were detected for the number of subjects taking stomach protectors and their prescription at baseline, and for the number of antihypertensive drugs prescribed at 6 months.

Twenty-three patients were diagnosed of generalized periodontitis in stage III and 12 of periodontitis in stage IV, with grades B or C.

At baseline, no statistically significant differences between groups for any demographic characteristics, CVD status or periodontitis diagnosis were observed.

### 3.3 Periodontal outcomes

At baseline, no significant differences between groups were observed for any periodontal variable (Table 2). Periodontal therapy improved all periodontal parameters in both treatment groups, with significant differences for mean PD and BoP reductions between baseline and 3 months, and baseline and 6 months (Supplementary Tables S2, S3).

**Table 2:** Periodontal outcomes.

Variable	Group	Baseline								3 Months								6 Months							
		*N*	Mean	SD	Mean diff.	95% CI		*p* value (*T*-Student)	*p* value (*U*-Mann Whitney)	*N*	Mean	SD	Mean diff.	95% CI		*p* value (*T*-Student)	*p* value (*U*-Mann Whitney)	*N*	Mean	SD	Mean diff.	95% CI		*p* value (*T*-Student)	*p* value (*U*-Mann Whitney)
						Lower	Upper							Lower	Upper							Lower	Upper		
PlI (%)	Control	19	83.76	12.42	0.86	−10.63	12.37	0.879		16	54.66	16.27	6.34	−11.54	24.22	0.467		17	53.50	20.82	−1.35	−16.71	14.01	0.858	
	Test	16	82.89	20.63						13	48.32	27.06						13	54.85	19.70					
REC (mm)	Control	19	1.09	0.98	0.16	−0.41	0.73	0.567		16	1.06	1.01	−0.03	−0.71	0.64	0.926		17	1.21	1.12	0.17	−0.50	0.85	0.604	
	Test	16	0.93	0.59						13	1.09	0.69						13	1.04	0.67					
PD (mm)	Control	19	4.28	0.59	0.34	−0.07	0.75	0.097		16	3.52	0.54	0.66*	0.28	1.04	0.001*		17	3.60	0.85	0.67*	0.19	1.15	0.008*	
	Test	16	3.93	0.59						13	2.87	0.43						13	2.93	0.37					
PD 1–3 mm (%)	Control	19	33.66	13.39	−0.08	−0.19	0.04	0.176		16	58.76	15.68	−0.21*	−0.31	−0.10	<0.001*		17	59.63	21.43	−0.20*	−0.32	−0.08	0.002*	
	Test	16	41.63	20.47						13	79.46	10.98						13	80.19	9.78					
PD 4–6 mm (%)	Control	19	48.8	12.14	−0.02	−0.08	0.12	0.712		16	31.11	11.05	0.14*	0.06	0.218	0.001*		17	27.72	14.53	0.11*	0.02	0.20	0.019*	
	Test	16	47.02	16.79						13	17.27	9.59						13	16.67	7.44					
PD >6 mm (%)	Control	19	17.49	11.56	0.06	−0.01	0.13	0.093		16	10.12	7.74	0.07*	0.02	0.11	0.005*		17	12.63	13.11	0.09*	0.02	0.16	0.01*	
	Test	16	11.34	8.97						13	3.25	3.71						13	3.13	3.31					
CAL (mm)	Control	19	5.37	1.46	0.50	−0.37	1.38	0.251		16	4.59	1.52	0.63	−0.35	1.61	0.200		17	4.81	1.87	0.84	−0.21	1.90	0.113	
	Test	16	4.86	1.00						13	3.96	0.89						13	3.97	0.84					
BOP (%)	Control	19	60.00	21.00	4.80	−7.13	16.72	0.419		16	45.04	17.35	18.75*	8.02	29.48	0.001*		17	46.79	21.21	18.58*	6.36	30.79	0.004*	
	Test	16	55.20	12.00						13	26.29	10.42						13	28.22	10.60					
SUP (%)	Control	19	1.63	3.80	0.74^a^	−1.36^a^	2.84^a^		0.806	16	0.49	0.93	0.21^a^	−0.35^a^	0.77^a^		0.983	17	2.50	7.09	2.50*^,^^a^	−1.54^a^	6.55^a^		0.002*
	Test	16	0.88	1.75						13	0.27	0.36						13	0.00	0.00					

SD, standard deviation; CI, confidence interval; PlI, plaque index; REC, recession; PD, pocket depth; CAL, clinical attachment level; BOP, bleeding on probing; SUP, suppuration.

* Statistically significant differences (*p* < 0.05).

^a^ Variables with non-normal distribution.

Periodontal parameters were significantly better in the test group, at 3 months, for PD and BoP, and at 6 months, for PD, BoP, and Sup. Frequency distribution of periodontal pockets showed a marked reduction of periodontal pockets ≥4 mm in test group at 3 and 6 months. Significantly differences were observed between groups in the frequency distribution of all categories of PD at 3 and 6 months, with greater percentage of periodontal pockets ≥4 mm in control group.

### 3.4 Cardiovascular outcomes

**Figure 3:**
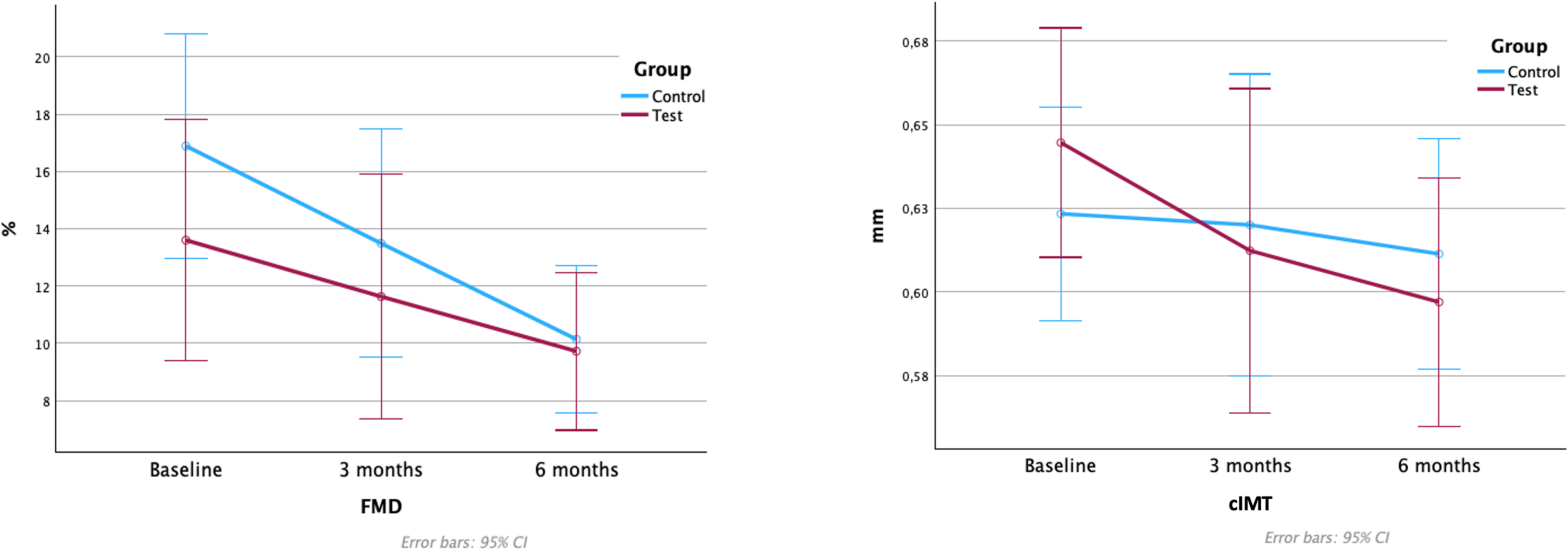
Flow-mediated dilation and carotid intima-media thickness values. FMD, flow-mediated dilation; cIMT, carotid intima-media thickness. Note: Figure based on data from the repeated measures ANOVA with *post hoc* Bonferroni's correction analysis.

Mean values and differences between groups in FMD and cIMT values are depicted in Table 3 and Figure 3.

**Table 3:** Surrogate measures of vascular function.

		Baseline								3 Months								6 Months							
Variable	Group	*N*	Mean	SD	Mean diff.	95% CI		*p* value (*T*-Student)	*p* value (*U*-Mann Whitney)	*N*	Mean	SD	Mean diff.	95% CI		*p* value (*T*-Student)	*p* value (*U*-Mann Whitney)	*N*	Mean	SD	Mean diff.	95% CI		*p* value (*T*-Student)	*p* value (*U*-Mann Whitney)
						Lower	Upper							Lower	Upper							Lower	Upper		
FMD (%)	Control	19	15.06	9.28	1.71	−3.35	6.77	0.495		16	13.74	7.10	2.94	−2.74	8.62	0.298		15	10.14	3.09	0.42	−3.63	4.46	0.831	
	Test	16	13.35	5.03						14	10.80	8.08						13	9.72	6.28					
cIMT (mm)	Control	19	0.63	0.05	−0.04	−0.08	0.01	0.131		16	0.62	0.06	−0.02	−0.10	0.06	0.590		15	0.61	0.07	0.01	−0.04	0.07	0.564	
	Test	16	0.67	0.08						14	0.64	0.15						13	0.60	0.06					

SD, standard deviation; CI, confidence interval; FMD, flow-mediated dilation; cIMT, carotid intima-media thickness.

A reduction in FMD was observed in both treatment groups, with statistically significant differences between baseline to 6 months in the control group [−6.75%, 95% CI (1.286; 12.217); *p* = 0.012] (Supplementary Table S4). At 3 months, the test group showed lower FMD, and at 6 months, the results were similar, with no significant differences between groups at any time-point.

No statistically significant differences in cIMT were detected between groups at any time point, with a tendency towards reductions in cIMT in both groups. The test group demonstrated significant reductions in cIMT from baseline to 6 months [−0.053 mm, 95% CI (0.009; 0.096); *p* = 0.014], while the corresponding changes were not statistically significant in the control group (Supplementary Table S5).

Table 4 shows the results of the lipid profile and blood pressure per visit. There were no statistically significant differences between groups at baseline or after 6 months. Subjects in the test group showed marked reductions in total cholesterol, triglycerides, HDL-C and LDL-C from baseline to 6 months, while in the control group these reductions were only observed for total cholesterol and LCL-C levels. There were no significant differences in BP between groups, with systolic and diastolic BP showing stable values in both groups during the 6 months follow-up.

**Table 4:** Lipids profile and blood pressure values.

		Baseline								6 Months							
Variable	Group	*N*	Mean	SD	Mean diff.	95% CI		*p* value (T-Student)	*p* value (U-Mann Whitney)	*N*	Mean	SD	Mean diff.	95% CI		*p* value (T-Student)	*p* value (U-Mann Whitney)
						Lower	Upper							Lower	Upper		
Lipids Profile																	
TC (mg/dl)	Control	19	138.16	33.47	0.91	−19.79	21.61	0.929		17	96.65	66.40	−10.05	−55.71	35.62	0.656	
	Test.	16	137.25	25.18						13	106.69	51.64					
HDL-C (mg/dl)	Control	19	45.79	12.31	1.66	−7.27	10.60	0.707		17	47.88	10.26	5.50	−2.90	13.90	0.191	
	Test	16	44.13	13.66						13	42.38	12.18					
LDL-C (mg/dl)	Control	19	72.26	28.12	5.39	−11.37	22.15	0.518		17	57.24	19.60	−3.23	−18.42	11.97	0.667	
	Test	16	66.88	18.64						13	60.46	20.82					
Triglycerides (mg/dl)	Control	19	97.32	49.52	33.03	−115.96	18.46	0.150		17	109.06	51.77	−12.71	−55.48	30.06	0.548	
	Test	16	146.06	133.83						13	121.77	62.62					
Blood Pressure																	
SBP (mmHg)	Control	19	130.95	14.49	−0.24	−10.88	10.40	0.964		17	132.94	19.64	3.02	−10.69	16.73	0.655	
	Test	16	131.19	16.47						13	129.92	15.99					
DBP (mmHg)	Control	19	82.67	7.26	1.00	−6.61	8.61	0.786		17	83.33	9.37	4.81	−4.80	14.42	0.307	
	Test	16	80.00	7.94						13	77.80	9.36					

SD, standard deviation; Diff., difference; CI, confidence interval; TC, total cholesterol; HDL-C, high-density lipoprotein cholesterol; LDL-C, low-density lipoprotein cholesterol; SBP, systolic blood pressure; DBP, diastolic blood pressure.

### 3.5 Serum inflammatory mediators and endothelium activation markers

**Figure 4:**
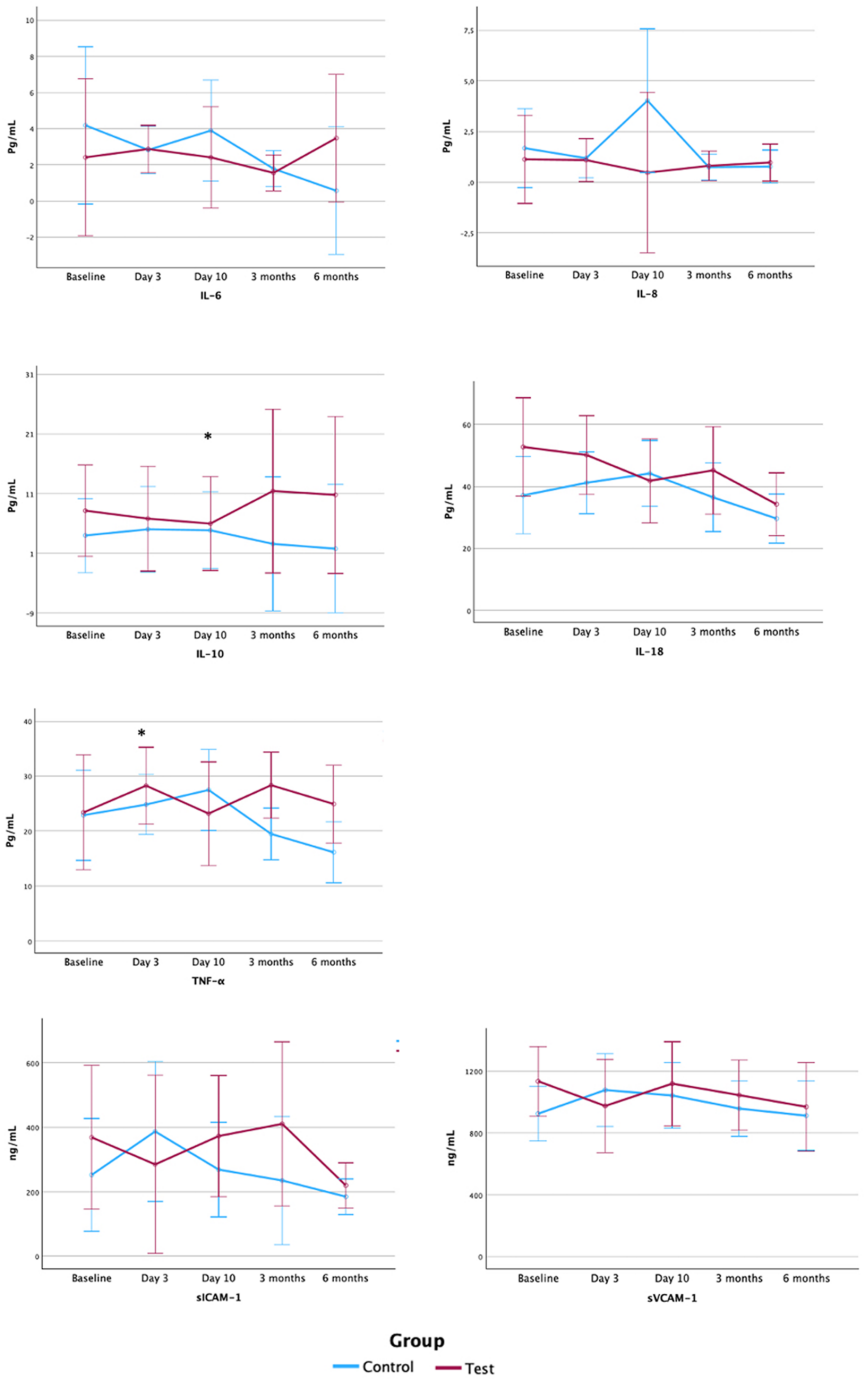
Serum levels of inflammatory mediators and endothelium activation markers. Pg, picogram; ml, millilitre; ng, nanogram; IL, interleukin; TNF, tumour necrosis factor; sICAM-1, soluble intercellular adhesion molecule-1; sVCAM-1, soluble vascular cell adhesion molecule 1; Gr., group. Note: Figure based on data from the repeated measures ANOVA with *post hoc* Bonferroni's correction analysis.

One hundred and fifty-nine samples were collected, and two of them could not be processed due to technical problems, thus a total of 157 samples were analyzed. Values under the detection limit of the kit were not included in the analysis. Results are shown in Table 5 and Figure 4.

**Table 5:** Serum levels of inflammatory mediators and endothelium activation markers.

		Baseline								3 Days								10 Days								3 Months								6 Months							
Variable	Group	*N*	Mean	SD	Mean diff.	95% CI		*p* value (*T*-Student)	*p* value (*U*-Mann Whitney)	*N*	Mean	SD	Mean diff.	95% CI		*p* value (*T*-Student)	*p* value (*U*-Mann Whitney)	*N*	Mean	SD	Mean diff.	95% CI		*p* value (*T*-Student)	*p* value (*U*-Mann Whitney)	*N*	Mean	SD	Mean diff.	95% CI		*p* value (*T*-Student)	*p* value (*U*-Mann Whitney)	*N*	Mean	SD	Mean diff.	95% CI		*p* value (*T*-Student)	*p* value (*U*-Mann Whitney)
						Lower	Upper							Lower	Upper							Lower	Upper							Lower	Upper							Lower	Upper		
IL−6 (Pg/ml)	Control	14	2.17	3.64	0.13^a^	−2.28^a^	2.54^a^		0.193	16	2.01	1.39	−0.84	−1.89	0.20	0.110		12	2.40	2.51	0.61	−1.04	2.25	0.454		8	1.42	1.16	−0.11	−1.10	0.88	0.811		16 10	0.70	0.45	−1.76	−4.81	1.29	0.237	
	Test	12	2.04	1.89						15	2.85	1.47						14	1.79	1.49						9	1.53	0.49							2.46	3.42					
IL−8 (Pg/ml)	Control	12	1.57	1.59	0.23	−1.11	1.57	0.722		15	1.15	0.86	0.20	−0.40	0.79	0.505		16	2.40	2.73	1.56*	0.05	3.06	0.043*		11	0.76	0.53	0.13	−0.31	0.57	0.543		14	0.77	0.64	−0.03	−0.66	0.60	0.922	
	Test	10	1.34	1.37						14	0.95	0.67						14	0.84	0.80						12	0.63	0.47						10	0.80	0.77					
IL−10 (Pg/ml)	Control	10	10.46	26.48	0.86	−27.35	32.33	0.859		10	9.47	22.05	6.27	−7.79	20.33	0.362		8	13.38	30.06	9.46	−15.27	34.18	0.424		7	1.70	0.86	−2.99	−8.16	2.18	0.216		5	2.70	3.03	−3.04	−10.77	4.70	0.392	
	Test	4	7.98	6.34						11	3.21	3.39						7	3.93	2.42						8	4.69	6.17						5	5.73	6.86					
IL−18 (Pg/ml)	Control	19	34.58	19.52	−19.64*	−36.92	−2.36	0.028*		19	34.49	18.02	−16.30*	−29.52	−3.07	0.017*		18	38.06	19.66	−5.65	−20.86	9.57	0.455		15	34.31	18.15	−6.53	−20.83	7.76	0.356		16	29.29	13.82	−9.16	−21.53	3.21	0.140	
	Test	15	54.23	27.42						16	50.79	20.43						15	43.71	23.22						13	40.85	18.59						11	38.45	17.36					
TNF-α (Pg/ml)	Control	19	22.65	15.73	−2.90	−12.54	6.75	0.545		19	23.89	10.32	−9.77	−23.63	4.10	0.157		18	26.41	13.69	2.42	−6.91	11.74	0.601		15	19.45	8.56	−5.83	−13.18	1.52	0.115		16	16.88	7.35	−6.92	−14.41	0.56	0.068	
	Test	15	25.55	10.56						16	33.66	24.78						15	23.99	12.31						13	25.28	10.37						11	23.80	11.59					
sICAM1 (ng/ml)	Control	19	280.97	241.72	−97.35	−369.76	175.07	0.465		19	327.52	324.82	−25.97	−312.91	260.96	0.855		18	257.99	148.68	−34.17^a^	−188.83^a^	120.49^a^		0.655	15	233.36	160.12	−87.31	−325.67	151.05	0.458		16	181.08	75.73	−35.32	−103.19	32.55	0.29	
	Test	15	378.32	458.06						16	353.49	503.46						15	292.16	278.10						13	320.67	415.92						11	216.40	95.37					
sVCAM1 (ng/ml)	Control	19	906.69	372.43	−78.26	−313.66	157.144	0.50		19	1,015.89	368.27	125.63	−120.85	372.11	0.310		18	1,008.37	385.55	62.92	−182.73	308.57	0.610		15	899.23	351.70	−84.78	−376.01	206.45	0.555		16	900.19	438.38	−14.15	−292.43	264.12	0.917	
	Test	15	984.95	278.51						16	890.26	343.07						15	945.46	286.91						13	984.01	398.23						11	914.34	261.54					

SD, standard deviation; CI, confidence interval; IL, interleukin; TNF, tumour necrosis factor; sICAM-1, soluble intercellular adhesion molecule-1; sVCAM-1, soluble vascular cell adhesion molecule 1, Pg, picogram; mL, millilitre; ng, nanogram.

* Statistically significant differences (*p* < 0.05).

^a^ Variables with non-normal distribution.

Since only four samples presented values of IL-1β above the detection limit of the kit, data on this inflammatory mediator is not presented. Statistically significant differences between groups in serum inflammatory markers were detected at baseline and 3 days for IL-18, with lower levels in control group, and at 10 days for IL-8, with lower values in test group. No further differences between groups were observed for the remaining inflammatory mediators or the endothelium activation markers, sICAM-1 and sVCAM-1.

### 3.6 Adverse events

No systemic adverse events were recorded. Minor to moderate bleeding was reported 12 h after periodontal therapy in 4 subjects in the test group, which was controlled by means of local haemostatic measures (gauze pressure, use of aminocaproic acid solution).

### 3.7 Post-hoc power and sample size calculation

Considering the reported results on the primary outcome (effect size of 2.87%, common SD of 8.14%), and assuming a two-sided alpha level of 5%, the power of the study resulted in 36%. Therefore, in order to obtain an 80% power, a total of 254 patients, 127 per group, would have been required (305 patients, considering a 20% more for drop-outs compensation and confounders adjustment).

## 4 Discussion

The results from the present investigation reveal that periodontal therapy (steps 1 and 2, with subgingival instrumentation and adjunctive antiseptics) of stage III–IV periodontitis, in subjects with recent history of acute coronary heart disease, provided some benefits in terms of cardiovascular and periodontal outcomes, although statistically significant differences with the control group were not observed, most probably due to the limited sample size. cIMT experienced a statistically significant reduction in test group from baseline to 6 months follow-up (−0.05 mm), while no statistically significant changes were observed for FMD (−3.45%). On its hand, control subjects experienced a slight worsening in their endothelial function, with statistically significant reductions in FMD from baseline to 6 months (−6.75%), while no changes were observed for cIMT values (−0.01 mm). These results should be interpreted with caution, since this pilot study was underpowered to detect significant differences between groups for the main outcome variable (36% post-hoc power calculation).

Previous investigations have explored the effect of periodontal therapy on endothelial function and systemic inflammation in different populations, with conflicting results. Tonetti and coworkers (19), in a RCT with 120 subjects with periodontitis and no history of CVD, reported improvements in FMD 6 months after “intensive” periodontal treatment (combining non-surgical therapy and systemic antibiotics), with statistically significant greater FMD in the “intensive” treatment group vs. the controls (absolute difference 2.0%). However, in periodontitis patients with established CVD, reported results on the effect of non-surgical periodontal treatment on 69 subjects with coronary artery disease and periodontitis, were not able to detect statistically significant differences in FMD between groups after a 3 months follow-up (test, 1.37%,; control, 1.39%) (24). In this study, similarly to the results from the present investigation, there were no changes in the serum markers of endothelium activation in the test group, while sVCAM-1 and sICAM-1 increased in controls, with significant differences between groups at 3 months.

Flow-mediated dilation (FMD) is a surrogate marker of CVD, widely used in intervention and epidemiological studies, since it is a non-invasive sensitive method to assess endothelial function. However, FMD has certain technical limitations related to its reproducibility and it is not frequently used in the clinical practice (25, 26). In systemically healthy subjects, mean FMD ranges between 0.20%–19.2%, while in coronary heart disease subjects FMD varies from −1.3%–14% (25). A cut-off value for normal endothelial function of 7.1% has been proposed (27), but this figure should be interpreted with caution since has been estimated based on a Japanese population, and variations among different populations have been described (25). Previous RCTs in periodontitis patients have reported mean FMD values of 6.5%–7.1% in cardiovascular healthy subjects (19) and 7.05%–7.10% in coronary heart disease patients (24). Our sample presented baseline values of FMD substantially higher (test, 13.35% vs. control, 15.06%), and despite a reduction in FMD in both groups after periodontal treatment, final values were still above the cut-off value estimated for normal endothelial function (test, 9.72% vs. control, 10.14%). These baseline FMD values in a population with established CVD could be the result of the compliance with multiple medications prescribed as part of their secondary prevention cardiovascular rehabilitation. Under these circumstances, the possible effect of periodontal therapy on FMD, although demonstrating improvements, could be confounded by the high values of FMD at baseline.

Carotid intima-media thickness is also a non-invasive surrogate marker of early detection of atherosclerosis widely used clinical trials in CVD (28), since it has been associated with several forms of CVD (29–31). A RCT on the effect of steps 1 and 2 of periodontal treatment vs. oral hygiene instructions alone on 169 Aboriginal Australians with periodontitis and no previous history of CVD, observed a statistically significant reduction in cIMT at 12 months, in the intervention group, of −0.023 mm, while no statistically significant differences were observed in controls (0.002 mm). However, as reported by the authors, this improvement did not translate in better endothelial function, measured through pulse wave velocity (20). Similarly, a cohort study on systemically healthy subjects, with mild to moderate forms of periodontitis, reported statistically significant reductions of −0.12 mm, 12 months after step 2 of periodontal treatment, but no control group was available (32). In our study, we observed a statistically significant reduction in cIMT of −0.05 mm in the test group after 6 months, with no significant differences between groups at any time point: thus, it was not possible to elucidate whether these changes were due to the periodontal therapy assigned or to the concomitant medical treatments.

Previous publications have reported a transient elevation of systemic inflammation, 24 hours after periodontal therapy, in subjects with and without CVD, that tend to disappear 7 days later, and subsequent improvements in these biomarker's serum levels in the long term (19, 32–36). This effect has been interpreted as a response to the “tissue trauma” induced by subgingival instrumentation, or as a possible systemic response to transient bacteraemia (37). This transient inflammatory response after therapy was observed in our investigation, in the test group, for TNF-α and IL-6, what may be explained by the tissue trauma during subgingival instrumentation, carried out only in the test group. However, we observed the previously reported tendency towards a reduction of serum levels in time for all the biomarkers evaluated in both treatment groups, except for IL-6 in the test group, although differences between groups were not statistically significant. Caution must be taken when interpreting these results, since certain interleukins, specifically IL-1β and IL-10, showed low values, frequently under the detection limit of the kit, and analyses were based on less than 10 samples (see Table 2, Supplementary Table S1). This lack of differences between groups could be due to the anti-inflammatory effects of regular medications taken by all the subjects in this RCT, as part of their CVD rehabilitation program. In fact, statins, which are the first drug of choice for the management of dyslipidaemia (38), due to their proven efficacy in reducing LDL cholesterol (39), have an anti-inflammatory effect. This effect has also been investigated when statins have been applied as adjunct host-modulator agents in the treatment of periodontitis (40). Similarly, certain lipid-lowering drugs have proven to induce significant reductions in cIMT (41–43). Furthermore, aspirin, which was also prescribed in 11% of our sample for its antithrombotic effect, has anti-inflammatory properties, and 23% of our patients used vasodilators, which may have also influenced the FMD results. Within this complex context, of multiple medication protocols combining anti-inflammatory and lipid-lowering drugs, medication intake may have confounded the possible effect of the periodontal interventions on systemic inflammation or endothelial function. Adjusting for the confounding effect of patient's medications to evaluate the effect of periodontal therapy on these patients would require much larger samples than those of this pilot clinical trial.

Our investigation has different limitations, one of them related to the intervention in the test group, since it was limited to steps 1 and 2 of periodontal treatment (18, 44), while participants presented generalised stage III and stage IV periodontitis. It is well documented that, in the presence of deep periodontal pockets (≥6 mm), periodontal treatment consisting of steps 1 and 2 might not be sufficient to arrest periodontal inflammation (18). In fact, even though there was a substantial improvement in the periodontal parameters after treatment, it failed to result in disease resolution and adequate management of inflammation in the test (steps 1 and 2) and control (step 1) patients, since both treatment groups exhibited residual pockets (PD >6 mm: control group 12.6% and test group 3.1%) and high levels of BoP (control group ≈ 47% and test group ≈ 28%), 6 months after therapy. Clinical endpoints of therapy were only achieved in two test patients at 3 months, and in one test patient at 6 months. Therefore, additional surgical therapy (step 3) might have been needed in the treatment of some of these patients. Providing full periodontal treatment, including step 3 of the therapy, would have led to better disease resolution and, hypothetically, greater improvements in endothelial function and serum biomarkers. Another limitation is that oral hygiene instructions and performance by the patients (step 1) could not be considered as a successful intervention in the present study, since both treatment groups exhibited high levels of plaque (test and control groups above 50%). Furthermore, longer follow-ups are desirable, since due to the natural history of atherosclerosis (3), changes in its progression are slow and take long time to be observed. Similarly, the occurrence of hard outcomes of CVD should be ideally investigated, but they require large cohorts of patients and longer follow-ups. However, the main limitation of this study was the sample size, since this trial was underpowered to detect the intended difference between groups. Even though screening was extended for 4 years, the study population was insufficient to provide an adequate pool of subjects for recruitment. Furthermore, the onset of Covid-19 pandemic and the implementation of lockdown and access restrictions to medical facilities, precluded from continuing patient screening and recruitment, therefore the final sample was limited to 35 patients. Additional RCTs, involving multiple research centres to overcome recruiting limitations, with longer follow-ups and reliable measures of CVD are needed, to further clarify the possible impact of periodontal therapy on CVD.

Despite the acknowledged limitations, the findings from the present trial support the recommendations from the European Federation of Periodontology and the World Heart Federation to provide periodontal treatment to patients with stablished cardiovascular disease and periodontitis as soon as its cardiovascular condition allows (45). In our sample, treatment of advanced forms of periodontitis (stages III and IV) three months after an acute cardiovascular event, proved to be safe, with minor post-intervention bleeding reported, and to induce certain improvement in surrogate markers of CVD.

## Data Availability

The raw data supporting the conclusions of this article will be made available by the authors, without undue reservation.
