# Supplementary material for: Effect of periodontal therapy on endothelial function and serum biomarkers in patients with periodontitis and established cardiovascular disease: a pilot study
Source: Front Oral Health. 2025 Feb 10;6:1488941. doi: 10.3389/froh.2025.1488941 (PMC11847872; doi:10.3389/froh.2025.1488941)
Supplement: Supplementary file 1 [file Table1.docx]

Supplementary Material

# Supplementary Data

## Laboratory assessments

Concentrations of inflammatory mediators (interleukin [IL]-1β, IL-6, IL-8, IL-10, IL-18 and tumour necrosis factor [TNF]-α) and endothelium activation markers (soluble intercellular adhesion molecule-1 [sICAM-1] and soluble vascular adhesion molecule 1 [sVCAM-1]) in serum were calculated using multiplex map human immunoassays (Millipore®, Cat. #HCVD2MAG-67K and #HCYTA-60K, Billerica, MA, USA) using a Luminex-200 System Unit with the XY platform (Luminex®, Oosterhout, The Netherlands). Microspheres for classification and reporter readings, and sheath fluid were obtained from Luminex® Corporation for proper calibration of the technique. Sensitivities were 1.6 pg/mL, 0.64 pg/mL, 0.64 pg/mL, 2.6 pg/mL, 0.64 pg/mL, 6.4 pg/mL, 0.085 ng/mL and 0.122 ng/mL for IL-1β, IL-6, IL-8, IL-10, IL-18, TNF-α, sICAM-1 and sVCAM-1, respectively. The results obtained were analysed with xPonent® software and were expressed in picograms per millilitre, for the inflammatory mediators, and nanograms per millilitre, for the endothelium activation markers.

# Supplementary Figures and Tables

## Supplementary Tables

**2.1.1 Supplementary Table 1.** Medications at baseline and 6 months.

|  | | **Baseline** | | | | **6 Months** | |  |  |
| --- | --- | --- | --- | --- | --- | --- | --- | --- | --- |
|  | **Test Group n=16** | | **Control Group n=19** | ***p value*** | **Test Group n=13** | | **Control Group n=17** | | ***p value*** |
| **Anticoagulant drugs *n (%)*** | 3 (18.8) | | 1 (5.3) | *0.312* | 3 (23.1) | | 1 (5.9) | | *0.170* |
| *Nº anticoagulant drugs n (%)* | 0 drugs: 13 (81.3) 1 drug: 3 (18.8) | | 0 drugs: 18 (94.7) 1 drug: 1 (5.3) | *0.474* | 0 drugs: 10 (76.9) 1 drug; 3 (23.1) | | 0 drugs: 16 (94.1) 1 drug: 1 (5.9) | | *0.290* |
| **Antiplatelet drugs *n (%)*** | 16 (100) | | 19 (100) | *-* | 11 (84.6) | | 16 (94.1) | | *0.565* |
| *Nº antiplatelet drugs n (%)* | 1 drug: 3 (18.8) 2 drugs: 13 (81.3) | | 1 drug: 2 (10.5) 2 drugs: 17 (89.5) | *0.489* | 0 drugs: 2 (15.4) 1 drug: 8 (61.5) 2 drugs: 3 (23.1) | | 0 drugs: 1 (5.9) 1 drug: 11 (64.7) 2 drugs: 5 (29.4) | | *0.675* |
| **Hypolipidemic drugs *n (%)*** | 16 (100) | | 19 (100) | *-* | 17 (100) | | 17 (100) | | *-* |
| *hypolipidemic drugs n (%)* | 1 drug: 11 (68.8) 2 drugs: 5 (31.3) | | 1 drug: 17 (89.5) 2 drugs: 2 (10.5) | *0.124* | 1 drug: 9 (69.2) 2 drugs: 3 (23.1) 3 drugs: 1 (7.7) | | 1 drug: 13 (76.5) 2 drugs: 3 (17.6) 3 drugs: 1 (5.9) | | *0.906* |
| **Antihypertensive drugs *n (%)*** | 16 (100) | | 19 (100) | *-* | 17 (100) | | 17 (100) | | *-* |
| *antihypertensive drugs n (%)* | 1 drug: 4 (25) 2 drugs: 10 (62.5) 3 drugs: 1 (6.3) 4 drugs: 1 (6.3) | | 1 drug: 9 (47.4) 2 drugs: 8 (42.1) 3 drugs: 2 (10.5) 4 drugs: - | *0.355* | 1 drug: 2 (15.4) 2 drugs: 10 (76.9) 3 drugs: - 4 drugs: 1 (7.7) | | 1 drug: 10 (58.8) 2 drugs: 5 (29.4) 3 drugs: 2 (11.8) 4 drugs: - | | *0.011** |
| **Vasodilator drugs *n (%)*** | 4 (25) | | 4 (21.1) | *0.782* | 3 (23.1) | | 3 (17.6) | | *1.000* |
| *vasodilator drugs n (%)* | 0 drugs: 12 (75) 1 drug; 4 (25) | | 0 drugs: 15 (78.9) 1 drug: 4 (21.1) | *0.259* | 0 drugs: 10 (76.9) 1 drug: 3 (23.1) | | 0 drugs: 14 (82.4) 1 drug: 3 (17.6) | | *1.000* |
| **Oral hypoglycemic drugs *n (%)*** | 2 (12.5) | | 3 (15.8) | *1.000* | 2 (15.4) | | 3 (17.6) | | *0.869* |
| *oral hypoglycemic drugs n (%)* | 0 drugs: 14 (87.5) 1 drug: 2 (12.5) | | 0 drugs: 16 (84.2) 1 drug: 3 (15.8) | *0.781* | 0 drugs: 11 (84.6) 1 drug: 2 (15.4) | | 0 drug: 14 (82.4) 3 (17.6) | | *1.000* |
| **Insuline *n (%)*** | 0 (0) | | 0 (0) | *-* | 0 (0) | | 0 (0) | | *-* |
| **Antidepressant drugs *n (%)*** | 1 (6.3) | | 2 (10.5) | *1.000* | 1 (13) | | 2 (11.8) | | *1.000* |
| *antidepressant drugs n (%)* | 0 drugs: 15 (93.8) 1 drug: -  2 drugs: 1 (6.3) 3 drugs: - | | 0 drugs: 17 (89.5) 1 drug: 1 (5.3) 2 drugs: -  3 drugs: 1 (5.3) | *0.649* | 0 drugs: 12 (92.3) 1 drug: - 2 drugs: 1 (7.7) | | 0 drugs: 15 (88.2) 1 drug: 2 (11.8) 2 drugs: - | | *0.138* |
| **Stomach protector *n (%)*** | 12 (75) | | 7 (36.8) | *0.024** | 10 (76.9) | | 7 (41.2) | | *0.050* |
| *stomach protector n (%)* | 0 drugs: 4 (25) 1 drug: 11 (68.8) 2 drugs: 1 (6.3) | | 0 drugs: 12 (63.2) 1 drug: 7 (36.8) 2 drugs: - | *0.045** | 0 drugs: 3 (23.1) 1 drug: 10 (76.9) | | 0 drug: 10 (58.8) 1 drugs: 7 (41.2) | | *0.050* |
| **Other drugs *n (%)*** | 3 (15.8) | | 5 (31.3) | *0.278* | 3 (17.6) | | 4 (30.8) | |  |

*n,* number.

* *Statistically significant differences (p < 0.05).*

**2.1.2 Supplementary Table 2.** Repeated measures ANOVA with post hoc Bonferroni's correction for pocket depth

| **Comparison** | **1st comparison** | **2nd comparison** | **n** | **Mean** | **SE** | **p-value** | **Mean Difference** | **95% CI of the difference** | | **p-value** | **Comparison** |  |
| --- | --- | --- | --- | --- | --- | --- | --- | --- | --- | --- | --- | --- |
|  |  |  |  |  |  |  |  | **Lower Bound** | **Upper Bound** |  |  |  |
| **Time** | ***Baseline*** |  | 27 | 4.13 | 0.10 | <0.001* | 0.93* | 0.76 | 1.10 | <0.001* | 1 VS 2 |  |
|  |  |  |  |  |  |  |  |  |  |  |  |  |
|  | ***3 months*** |  | 27 | 3.19 | 0.10 |  | 0.93* | 0.76 | 1.10 | <0.001* | 1 VS 3 |  |
|  |  |  |  |  |  |  |  |  |  |  |  |  |
|  | ***6 months*** |  | 27 | 3.20 | 0.11 |  | -0.01 | -0.17 | 0.15 | 1.000 | 2 VS 3 |  |
|  |  |  |  |  |  |  |  |  |  |  |  |  |
| **Group** | ***Control*** |  | 15 | 3.76 | 0.12 | 0.010* | 0.51* | 0.13 | 0.89 | 0.010* | Control vs Test |  |
|  | ***Test*** |  | 12 |  |  |  |  |  |  |  |  |  |
| **Group*Time** | ***Control*** | ***Baseline*** | 15 | 4.26 | 0.13 | 0.030* | 0.72* | 0.49 | 0.95 | <0.001* | 1 VS 2 |  |
|  |  |  |  |  |  |  |  |  |  |  |  |  |
|  |  | ***3 months*** | 15 | 3.54 | 0.13 |  | 0.77* | 0.45 | 1.10 | <0.001* | 1 VS 3 |  |
|  |  |  |  |  |  |  |  |  |  |  |  |  |
|  |  | ***6 months*** | 15 | 3.49 | 0.14 |  | 0.06 | -0.16 | 0.27 | 1.000 | 2 VS 3 |  |
|  |  |  |  |  |  |  |  |  |  |  |  |  |
|  | ***Test*** | ***Baseline*** | 12 | 3.99 | 0.15 |  | 1.15* | 0.89 | 1.41 | <0.001* | 1 VS 2 |  |
|  |  |  |  |  |  |  |  |  |  |  |  |  |
|  |  | ***3 months*** | 12 | 2.85 | 0.15 |  | 1.07* | 0.71 | 1.44 | <0.001* | 1 VS 3 |  |
|  |  |  |  |  |  |  |  |  |  |  |  |  |
|  |  | ***6 months*** | 12 | 2.92 | 0.16 |  | -0.08 | -0.31 | 0.16 | 1.000 | 2 VS 3 |  |
|  |  |  |  |  |  |  |  |  |  |  |  |  |
|  | ***Baseline*** | ***Control*** | 15 | 4.26 | 0.13 |  | 0.26 | -0.14 | 0.67 | 0.190 | Control vs Test |  |
|  |  | ***Test*** | 12 |  |  |  |  |  |  |  |  |  |
|  | ***3 months*** | ***Control*** | 15 | 3.54 | 0.13 |  | 0.70* | 0.29 | 1.10 | <0.001* | Control vs Test |  |
|  |  | ***Test*** | 12 |  |  |  |  |  |  |  |  |  |
|  | ***6 months*** | ***Control*** | 15 | 3.49 | 0.14 |  | 0.57* | 0.13 | 1.01 | 0.010* | Control vs Test |  |
|  |  | ***Test*** | 12 |  |  |  |  |  |  |  |  |  |

*n,* number; *SE,* standard error; *CI,* confidence interval.

* *Statistically significant differences (p < 0.05).*

**2.1.3 Supplementary Table 3.** Repeated measures ANOVA with post hoc Bonferroni's correction for bleeding on probing

| **Comparison** | **1st comparison** | **2nd comparison** | **n** | **Mean** | **SE** | **p-value** | **Mean Difference** | **95% CI of the difference** | | **p-value** | **Comparison** |  |
| --- | --- | --- | --- | --- | --- | --- | --- | --- | --- | --- | --- | --- |
|  |  |  |  |  |  |  |  | **Lower Bound** | **Upper Bound** |  |  |  |
| **Time** | ***Baseline*** |  | 27 | 0.59 | 0.03 | <0.001* | 0.23* | 0.15 | 0.30 | <0.001* | 1 VS 2 |  |
|  |  |  |  |  |  |  |  |  |  |  |  |  |
|  | ***3 months*** |  | 27 | 0.36 | 0.03 |  | 0.22* | 0.14 | 0.31 | <0.001* | 1 VS 3 |  |
|  |  |  |  |  |  |  |  |  |  |  |  |  |
|  | ***6 months*** |  | 27 | 0.37 | 0.03 |  | -0.00 | -0.07 | 0.06 | 1.000 | 2 VS 3 |  |
|  |  |  |  |  |  |  |  |  |  |  |  |  |
| **Group** | ***Control*** |  | 15 | 0.51 | 0.04 | 0.014* | 0.14* | 0.03 | 0.25 | 0.014* | Control vs Test |  |
|  | ***Test*** |  | 12 |  |  |  |  |  |  |  |  |  |
| **Group*Time** | ***Control*** | ***Baseline*** | 15 | 0.61 | 0.05 | 0.013* | 0.15* | 0.05 | 0.25 | 0.002* | 1 VS 2 |  |
|  |  |  |  |  |  |  |  |  |  |  |  |  |
|  |  | ***3 months*** | 15 | 0.46 | 0.04 |  | 0.15* | 0.03 | 0.26 | 0.009* | 1 VS 3 |  |
|  |  |  |  |  |  |  |  |  |  |  |  |  |
|  |  | ***6 months*** | 15 | 0.46 | 0.04 |  | 0.00 | -0.08 | 0.08 | 1.000 | 2 VS 3 |  |
|  |  |  |  |  |  |  |  |  |  |  |  |  |
|  | ***Test*** | ***Baseline*** | 12 | 0.57 | 0.05 |  | 0.31* | 0.20 | 0.42 | <0.001* | 1 VS 2 |  |
|  |  |  |  |  |  |  |  |  |  |  |  |  |
|  |  | ***3 months*** | 12 | 0.26 | 0.04 |  | 0.30* | 0.17 | 0.43 | <0.001* | 1 VS 3 |  |
|  |  |  |  |  |  |  |  |  |  |  |  |  |
|  |  | ***6 months*** | 12 | 0.27 | 0.05 |  | -0.01 | -0.10 | 0.08 | 1.000 | 2 VS 3 |  |
|  |  |  |  |  |  |  |  |  |  |  |  |  |
|  | ***Baseline*** | ***Control*** | 15 | 0.61 | 0.05 |  | 0.04 | -0.10 | 0.18 | 0.571 | Control vs Test |  |
|  |  | ***Test*** | 12 | 0.57 | 0.05 |  |  |  |  |  |  |  |
|  | ***3 months*** | ***Control*** | 15 | 0.46 | 0.04 |  | 0.20* | 0.08 | 0.32 | 0.002* | Control vs Test |  |
|  |  | ***Test*** | 12 | 0.26 | 0.04 |  |  |  |  |  |  |  |
|  | ***6 months*** | ***Control*** | 15 | 0.46 | 0.04 |  | 0.19* | 0.06 | 0.32 | 0.006* | Control vs Test |  |
|  |  | ***Test*** | 12 | 0.27 | 0.05 |  |  |  |  |  |  |  |

*n,* number; *SE,* standard error; *CI* confidence interval.

* *Statistically significant differences (p < 0.05).*

**2.1.4 Supplementary Table 4.** Repeated measures ANOVA with post hoc Bonferroni's correction for flow-mediated dilation

| **Comparison** | **1st comparison** | **2nd comparison** | **n** | **Mean** | **SE** | **p-value** | **Mean Difference** | **95% CI of the difference** | | **p-value** | **Comparison** |  |
| --- | --- | --- | --- | --- | --- | --- | --- | --- | --- | --- | --- | --- |
|  |  |  |  |  |  |  |  | **Lower Bound** | **Upper Bound** |  |  |  |
| **Time** | ***Baseline*** |  | 27 | 15.21 | 1.46 | 0.031* | 2.47 | -3.19 | 8.12 | 0.819 | 1 VS 2 |  |
|  |  |  |  |  |  |  |  |  |  |  |  |  |
|  | ***3 months*** |  | 27 | 12.74 | 1.47 |  | 5.09* | 0.99 | 9.19 | 0.012* | 1 VS 3 |  |
|  |  |  |  |  |  |  |  |  |  |  |  |  |
|  | ***6 months*** |  | 27 | 10.12 | 0.94 |  | 2.62 | -1.87 | 7.11 | 0.439 | 2 VS 3 |  |
|  |  |  |  |  |  |  |  |  |  |  |  |  |
| **Group** | ***Control*** |  | 15 | 13.50 | 1.00 | 0.291 | 1.62 | -1.47 | 4.71 | 0.291 | Control vs Test |  |
|  | ***Test*** |  | 12 | 11.89 | 1.12 |  |  |  |  |  |  |  |
| **Group*Time** | ***Control*** | ***Baseline*** | 15 | 16.89 | 1.95 | 0.644 | 3.40 | -4.14 | 10.94 | 0.774 | 1 VS 2 |  |
|  |  |  |  |  |  |  |  |  |  |  |  |  |
|  |  | ***3 months*** | 15 | 13.49 | 1.96 |  | 6.75* | 1.29 | 12.22 | 0.012* | 1 VS 3 |  |
|  |  |  |  |  |  |  |  |  |  |  |  |  |
|  |  | ***6 months*** | 15 | 10.14 | 1.25 |  | 3.35 | -2.63 | 9.33 | 0.489 | 2 VS 3 |  |
|  |  |  |  |  |  |  |  |  |  |  |  |  |
|  | ***Test*** | ***Baseline*** | 12 | 13.54 | 2.18 |  | 1.54 | -6.89 | 9.97 | 1.000 | 1 VS 2 |  |
|  |  |  |  |  |  |  |  |  |  |  |  |  |
|  |  | ***3 months*** | 12 | 12.00 | 2.19 |  | 3.43 | -2.68 | 9.54 | 0.487 | 1 VS 3 |  |
|  |  |  |  |  |  |  |  |  |  |  |  |  |
|  |  | ***6 months*** | 12 | 10.11 | 1.39 |  | 1.89 | -4.80 | 8.58 | 1.000 | 2 VS 3 |  |
|  |  |  |  |  |  |  |  |  |  |  |  |  |
|  | ***Baseline*** | ***Control*** | 15 | 0.62 | 0.02 |  | 3.35 | -2.67 | 9.36 | 0.260 | Control vs Test |  |
|  |  | ***Test*** | 12 | 0.65 | 0.02 |  |  |  |  |  |  |  |
|  | ***3 months*** | ***Control*** | 15 | 0.62 | 0.02 |  | 1.48 | -4.58 | 7.54 | 0.620 | Control vs Test |  |
|  |  | ***Test*** | 12 | 0.61 | 0.03 |  |  |  |  |  |  |  |
|  | ***6 months*** | ***Control*** | 15 | 0.61 | 0.02 |  | 0.02 | -3.83 | 3.88 | 0.990 | Control vs Test |  |
|  |  | ***Test*** | 12 | 0.59 | 0.02 |  |  |  |  |  |  |  |

*n* number; *SE* standard error; *CI* confidence interval.

* *Statistically significant differences (p < 0.05).*

**2.1.5 Supplementary Table 5.** Repeated measures ANOVA with post hoc Bonferroni's correction for cIMT

| **Comparison** | **1st comparison** | **2nd comparison** | **n** | **Mean** | **SE** | **p-value** | **Mean Difference** | **95% CI of the difference** | | **p-value** | **Comparison** |  |
| --- | --- | --- | --- | --- | --- | --- | --- | --- | --- | --- | --- | --- |
|  |  |  |  |  |  |  |  | **Lower Bound** | **Upper Bound** |  |  |  |
| **Time** | ***Baseline*** |  | 27 | 0.63 | 0.01 | 0.012* | 0.02 | -0.01 | 0.05 | 0.183 | 1 VS 2 |  |
|  |  |  |  |  |  |  |  |  |  |  |  |  |
|  | ***3 months*** |  | 27 | 0.61 | 0.02 |  | 0.03* | 0.00 | 0.06 | 0.025* | 1 VS 3 |  |
|  |  |  |  |  |  |  |  |  |  |  |  |  |
|  | ***6 months*** |  | 27 | 0.60 | 0.01 |  | 0.01 | -0.01 | 0.04 | 0.735 | 2 VS 3 |  |
|  |  |  |  |  |  |  |  |  |  |  |  |  |
| **Group** | ***Control*** |  | 15 | 0.62  0.62 | 0.02  0.02 | 0.916 | 0.00 | -0.05 | 0.05 | 0.916 | Control vs Test |  |
|  | ***Test*** |  | 12 |  |  |  |  |  |  |  |  |  |
| **Group*Time** | ***Control*** | ***Baseline*** | 15 | 0.62 | 0.02 | 0.124 | 0.00 | -0.03 | 0.04 | 1.000 | 1 VS 2 |  |
|  |  |  |  |  |  |  |  |  |  |  |  |  |
|  |  | ***3 months*** | 15 | 0.62 | 0.02 |  | 0.01 | -0.03 | 0.05 | 1.000 | 1 VS 3 |  |
|  |  |  |  |  |  |  |  |  |  |  |  |  |
|  |  | ***6 months*** | 15 | 0.61 | 0.02 |  | 0.01 | -0.02 | 0.04 | 1.000 | 2 VS 3 |  |
|  |  |  |  |  |  |  |  |  |  |  |  |  |
|  | ***Test*** | ***Baseline*** | 12 | 0.65 | 0.02 |  | 0.04 | -0.00 | 0.08 | 0.069 | 1 VS 2 |  |
|  |  |  |  |  |  |  |  |  |  |  |  |  |
|  |  | ***3 months*** | 12 | 0.61 | 0.02 |  | 0.05* | 0.01 | 0.10 | 0.014* | 1 VS 3 |  |
|  |  |  |  |  |  |  |  |  |  |  |  |  |
|  |  | ***6 months*** | 12 | 0.59 | 0.02 |  | 0.01 | -0.02 | 0.05 | 0.993 | 2 VS 3 |  |
|  |  |  |  |  |  |  |  |  |  |  |  |  |
|  | ***Baseline*** | ***Control*** | 15 | 0.62 | 0.02 |  | -0.02 | -0.03 | 0.07 | 0.350 | Control vs Test |  |
|  |  | ***Test*** | 12 | 0.65 | 0.02 |  |  |  |  |  |  |  |
|  | ***3 months*** | ***Control*** | 15 | 0.62 | 0.02 |  | 0.01 | -0.06 | 0.08 | 0.710 | Control vs Test |  |
|  |  | ***Test*** | 12 | 0.61 | 0.03 |  |  |  |  |  |  |  |
|  | ***6 months*** | ***Control*** | 15 | 0.61 | 0.02 |  | 0.02 | -0.03 | 0.07 | 0.490 | Control vs Test |  |
|  |  | ***Test*** | 12 | 0.59 | 0.02 |  |  |  |  |  |  |  |

*n,* number; *SE,* standard error; *CI,* confidence interval.

* *Statistically significant differences (p < 0.05*
